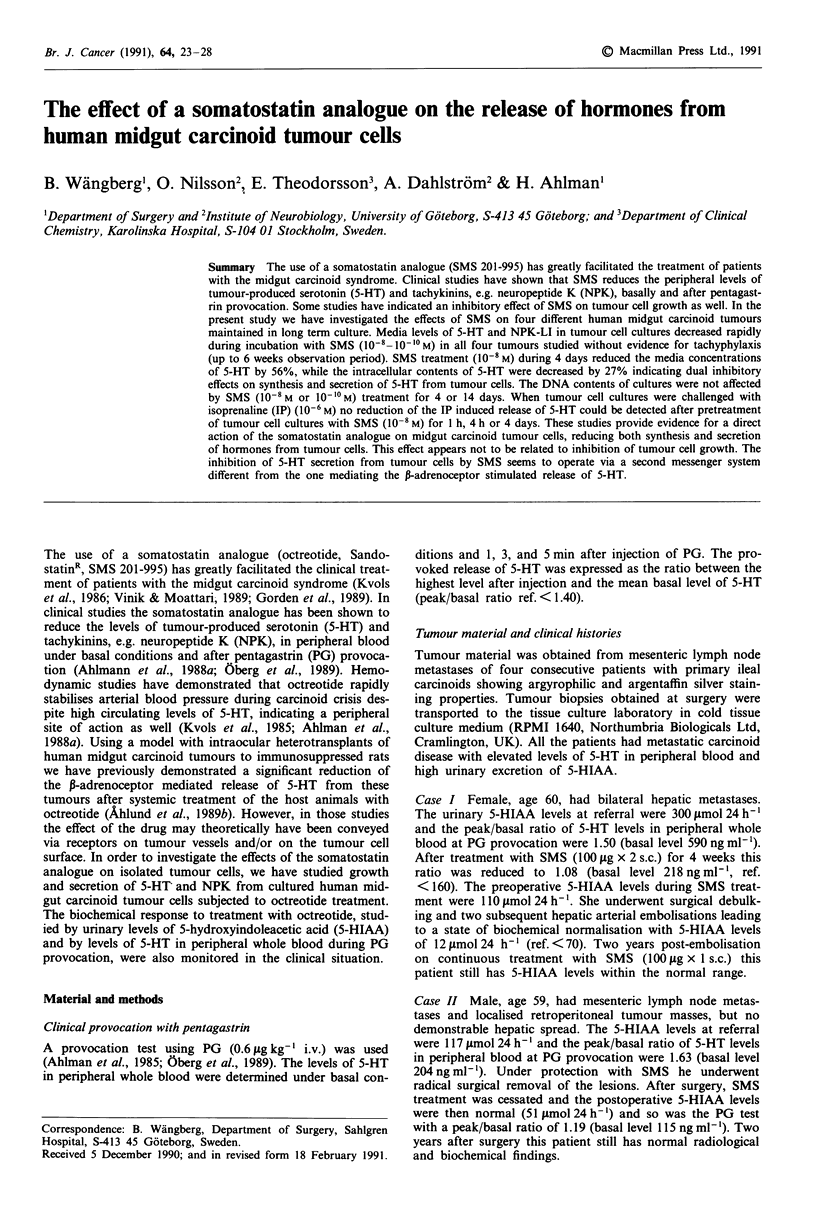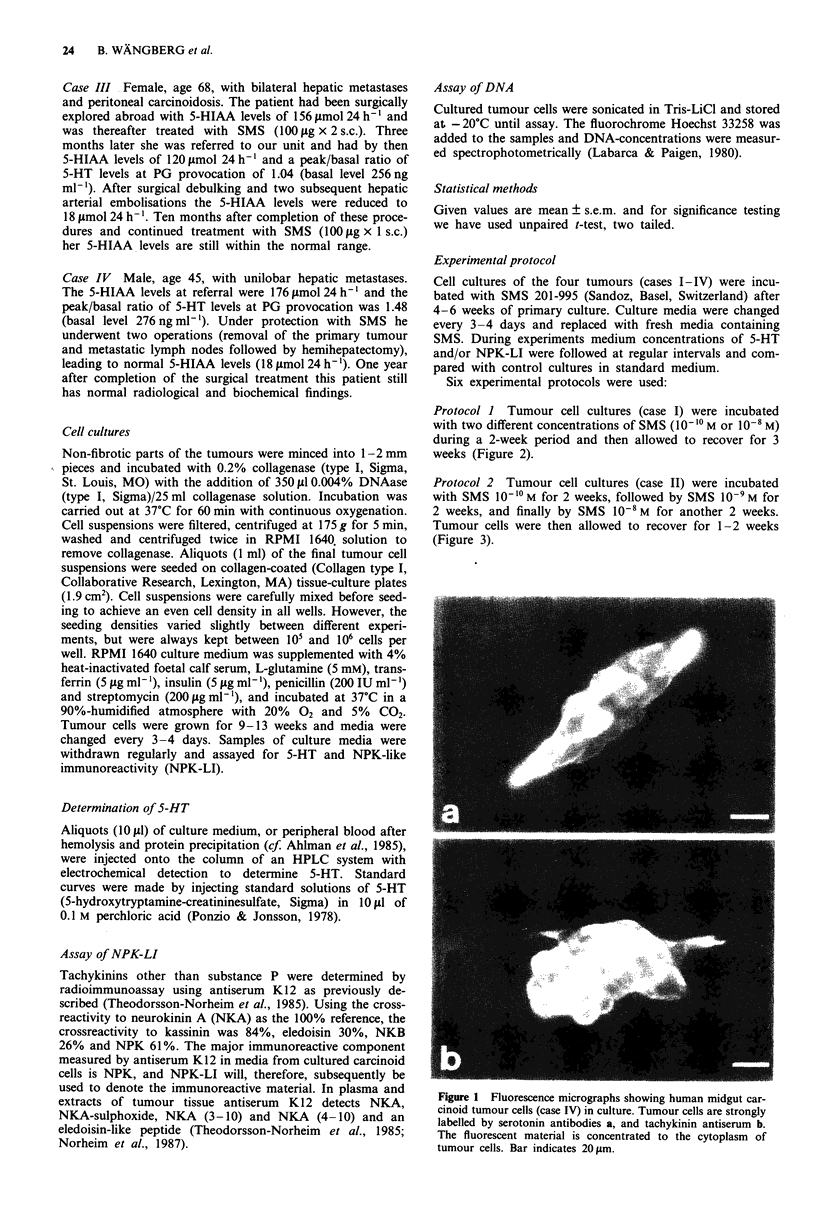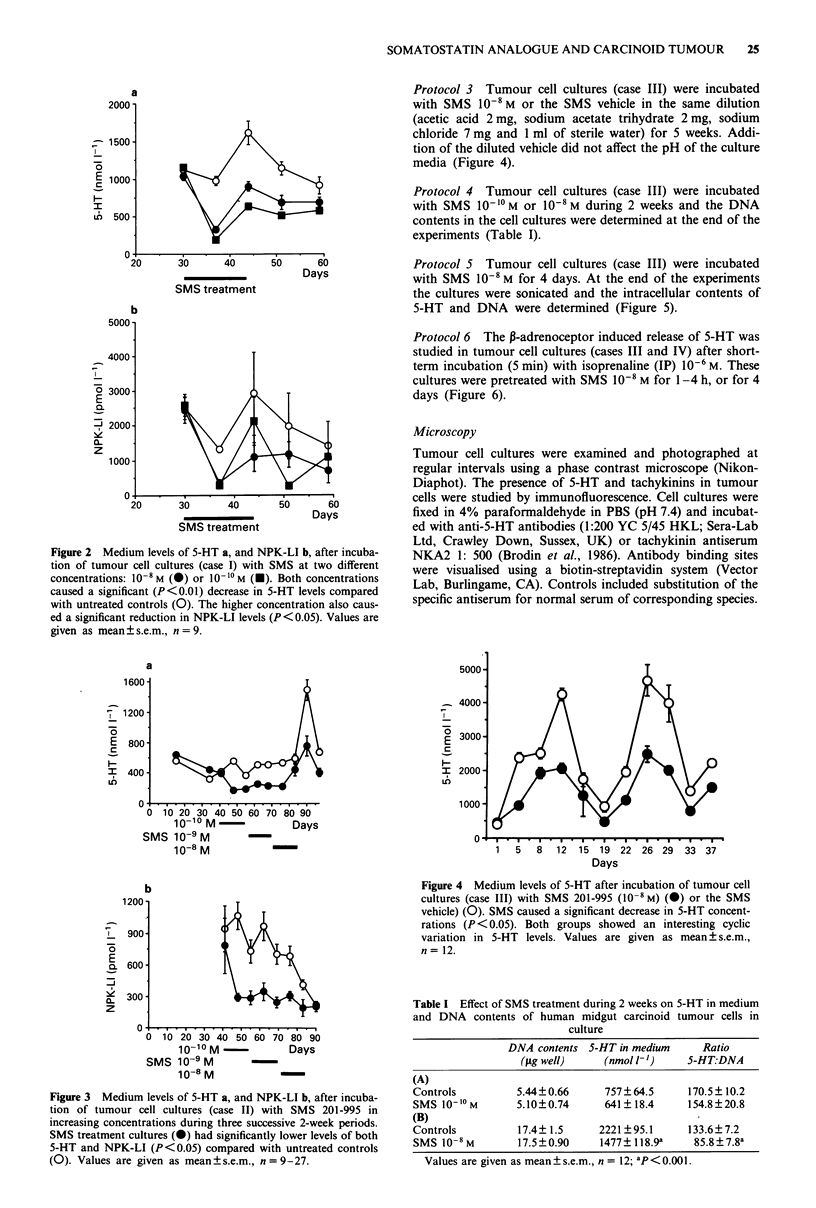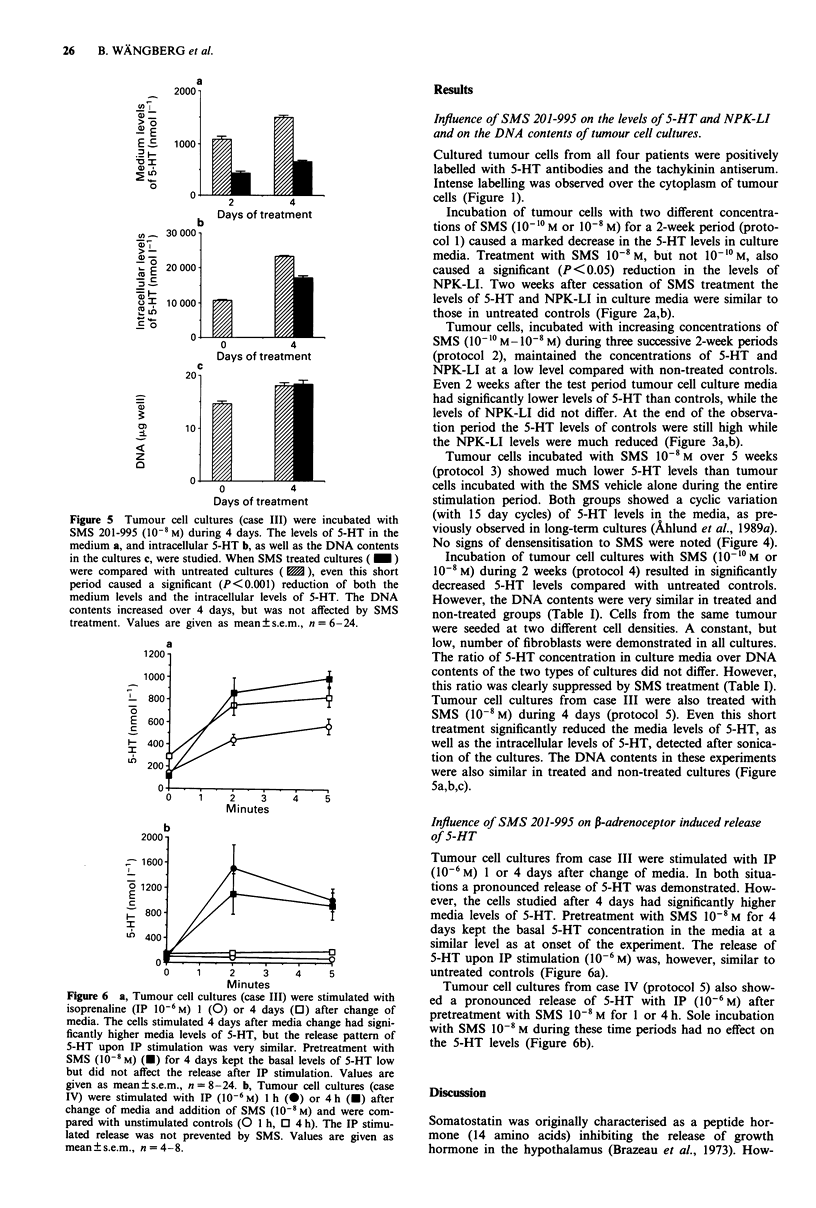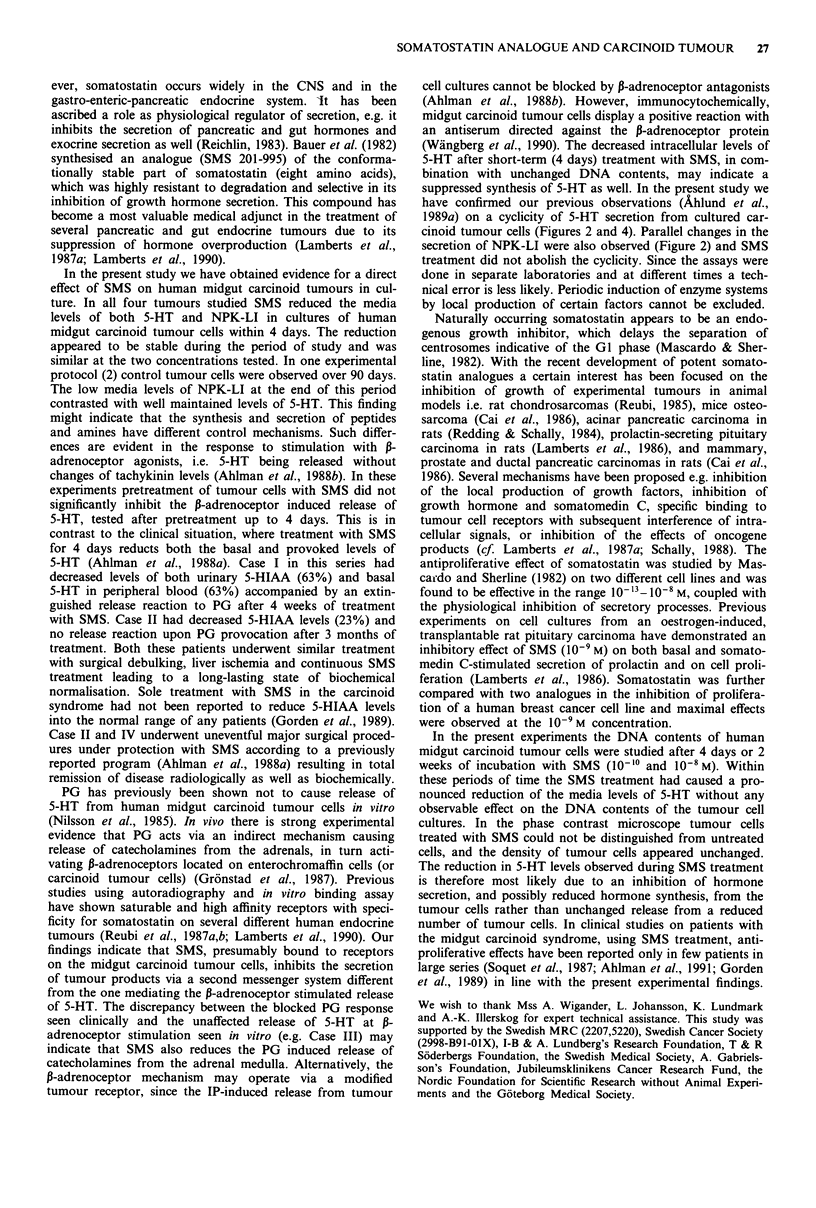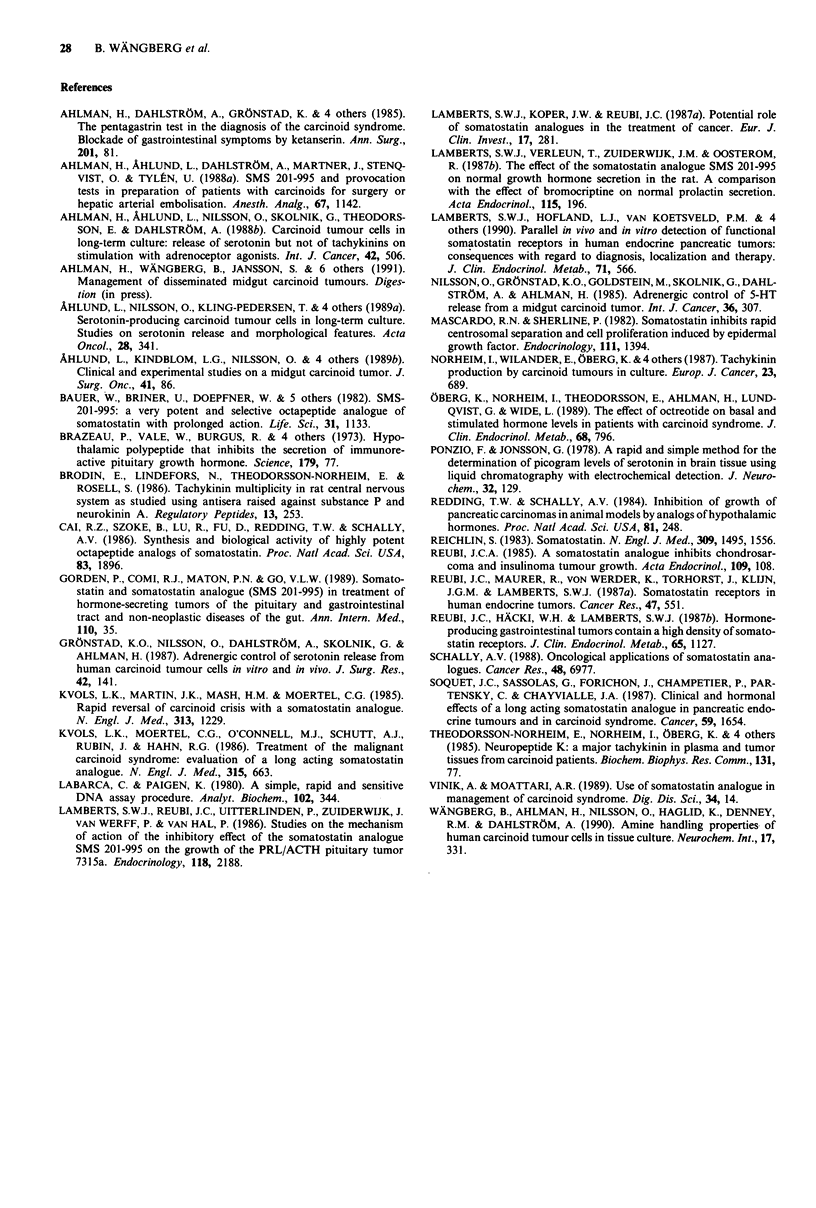# The effect of a somatostatin analogue on the release of hormones from human midgut carcinoid tumour cells.

**DOI:** 10.1038/bjc.1991.233

**Published:** 1991-07

**Authors:** B. Wängberg, O. Nilsson, E. Theodorsson, A. Dahlström, H. Ahlman

**Affiliations:** Department of Surgery, University of Göteborg, Sweden.

## Abstract

**Images:**


Br. J. Cancer (1991), 64, 23–28 © Macmillan Press Ltd., 1991

### The effect of a somatostatin analogue on the release of hormones from human midgut carcinoid tumour cells

B. Wängberg[1], O. Nilsson[2], E. Theodorsson[3], A. Dahlström[2] & H. Ahlman[1]

[1]*Department of Surgery and* [2]*Institute of Neurobiology, University of Göteborg, S-413 45 Göteborg; and* [3]*Department of Clinical Chemistry, Karolinska Hospital, S-104 01 Stockholm, Sweden.*

**Summary** The use of a somatostatin analogue (SMS 201-995) has greatly facilitated the treatment of patients with the midgut carcinoid syndrome. Clinical studies have shown that SMS reduces the peripheral levels of tumour-produced serotonin (5-HT) and tachykinins, e.g. neuropeptide K (NPK), basally and after pentagastrin provocation. Some studies have indicated an inhibitory effect of SMS on tumour cell growth as well. In the present study we have investigated the effects of SMS on four different human midgut carcinoid tumours maintained in long term culture. Media levels of 5-HT and NPK-LI in tumour cell cultures decreased rapidly during incubation with SMS ($10^{-8}$–$10^{-10}$ M) in all four tumours studied without evidence for tachyphylaxis (up to 6 weeks observation period). SMS treatment ($10^{-8}$ M) during 4 days reduced the media concentrations of 5-HT by 56%, while the intracellular contents of 5-HT were decreased by 27% indicating dual inhibitory effects on synthesis and secretion of 5-HT from tumour cells. The DNA contents of cultures were not affected by SMS ($10^{-8}$ M or $10^{-10}$ M) treatment for 4 or 14 days. When tumour cell cultures were challenged with isoprenaline (IP) ($10^{-6}$ M) no reduction of the IP induced release of 5-HT could be detected after pretreatment of tumour cell cultures with SMS ($10^{-8}$ M) for 1 h, 4 h or 4 days. These studies provide evidence for a direct action of the somatostatin analogue on midgut carcinoid tumour cells, reducing both synthesis and secretion of hormones from tumour cells. This effect appears not to be related to inhibition of tumour cell growth. The inhibition of 5-HT secretion from tumour cells by SMS seems to operate via a second messenger system different from the one mediating the β-adrenoceptor stimulated release of 5-HT.

The use of a somatostatin analogue (octreotide, Sandostatin[R], SMS 201-995) has greatly facilitated the clinical treatment of patients with the midgut carcinoid syndrome (Kvols *et al.*, 1986; Vinik & Moattari, 1989; Gorden *et al.*, 1989). In clinical studies the somatostatin analogue has been shown to reduce the levels of tumour-produced serotonin (5-HT) and tachykinins, e.g. neuropeptide K (NPK), in peripheral blood under basal conditions and after pentagastrin (PG) provocation (Ahlmann *et al.*, 1988*a*; Öberg *et al.*, 1989). Hemodynamic studies have demonstrated that octreotide rapidly stabilises arterial blood pressure during carcinoid crisis despite high circulating levels of 5-HT, indicating a peripheral site of action as well (Kvols *et al.*, 1985; Ahlman *et al.*, 1988*a*). Using a model with intraocular heterotransplants of human midgut carcinoid tumours to immunosuppressed rats we have previously demonstrated a significant reduction of the β-adrenoceptor mediated release of 5-HT from these tumours after systemic treatment of the host animals with octreotide (Åhlund *et al.*, 1989*b*). However, in those studies the effect of the drug may theoretically have been conveyed via receptors on tumour vessels and/or on the tumour cell surface. In order to investigate the effects of the somatostatin analogue on isolated tumour cells, we have studied growth and secretion of 5-HT and NPK from cultured human midgut carcinoid tumour cells subjected to octreotide treatment. The biochemical response to treatment with octreotide, studied by urinary levels of 5-hydroxyindoleacetic acid (5-HIAA) and by levels of 5-HT in peripheral whole blood during PG provocation, were also monitored in the clinical situation.

### Material and methods

### *Clinical provocation with pentagastrin*

A provocation test using PG (0.6 μg kg$^{-1}$ i.v.) was used (Ahlman *et al.*, 1985; Öberg *et al.*, 1989). The levels of 5-HT in peripheral whole blood were determined under basal conditions and 1, 3, and 5 min after injection of PG. The provoked release of 5-HT was expressed as the ratio between the highest level after injection and the mean basal level of 5-HT (peak/basal ratio ref. < 1.40).

### *Tumour material and clinical histories*

Tumour material was obtained from mesenteric lymph node metastases of four consecutive patients with primary ileal carcinoids showing argyrophilic and argentaffin silver staining properties. Tumour biopsies obtained at surgery were transported to the tissue culture laboratory in cold tissue culture medium (RPMI 1640, Northumbria Biologicals Ltd, Cramlington, UK). All the patients had metastatic carcinoid disease with elevated levels of 5-HT in peripheral blood and high urinary excretion of 5-HIAA.

*Case I* Female, age 60, had bilateral hepatic metastases. The urinary 5-HIAA levels at referral were 300 μmol 24 h$^{-1}$ and the peak/basal ratio of 5-HT levels in peripheral whole blood at PG provocation were 1.50 (basal level 590 ng ml$^{-1}$). After treatment with SMS (100 μg × 2 s.c.) for 4 weeks this ratio was reduced to 1.08 (basal level 218 ng ml$^{-1}$, ref. < 160). The preoperative 5-HIAA levels during SMS treatment were 110 μmol 24 h$^{-1}$. She underwent surgical debulking and two subsequent hepatic arterial embolisations leading to a state of biochemical normalisation with 5-HIAA levels of 12 μmol 24 h$^{-1}$ (ref. < 70). Two years post-embolisation on continuous treatment with SMS (100 μg × 1 s.c.) this patient still has 5-HIAA levels within the normal range.

*Case II* Male, age 59, had mesenteric lymph node metastases and localised retroperitoneal tumour masses, but no demonstrable hepatic spread. The 5-HIAA levels at referral were 117 μmol 24 h$^{-1}$ and the peak/basal ratio of 5-HT levels in peripheral blood at PG provocation were 1.63 (basal level 204 ng ml$^{-1}$). Under protection with SMS he underwent radical surgical removal of the lesions. After surgery, SMS treatment was cessated and the postoperative 5-HIAA levels were then normal (51 μmol 24 h$^{-1}$) and so was the PG test with a peak/basal ratio of 1.19 (basal level 115 ng ml$^{-1}$). Two years after surgery this patient still has normal radiological and biochemical findings.

Correspondence: B. Wängberg, Department of Surgery, Sahlgren Hospital, S-413 45 Göteborg, Sweden.
Received 5 December 1990; and in revised form 18 February 1991.

*Case III* Female, age 68, with bilateral hepatic metastases and peritoneal carcinoidosis. The patient had been surgically explored abroad with 5-HIAA levels of 156 μmol 24 $h^{-1}$ and was thereafter treated with SMS (100 μg × 2 s.c.). Three months later she was referred to our unit and had by then 5-HIAA levels of 120 μmol 24 $h^{-1}$ and a peak/basal ratio of 5-HT levels at PG provocation of 1.04 (basal level 256 ng $ml^{-1}$). After surgical debulking and two subsequent hepatic arterial embolisations the 5-HIAA levels were reduced to 18 μmol 24 $h^{-1}$. Ten months after completion of these procedures and continued treatment with SMS (100 μg × 1 s.c.) her 5-HIAA levels are still within the normal range.

*Case IV* Male, age 45, with unilobar hepatic metastases. The 5-HIAA levels at referral were 176 μmol 24 $h^{-1}$ and the peak/basal ratio of 5-HT levels at PG provocation was 1.48 (basal level 276 ng $ml^{-1}$). Under protection with SMS he underwent two operations (removal of the primary tumour and metastatic lymph nodes followed by hemihepatectomy), leading to normal 5-HIAA levels (18 μmol 24 $h^{-1}$). One year after completion of the surgical treatment this patient still has normal radiological and biochemical findings.

*Cell cultures*

Non-fibrotic parts of the tumours were minced into 1–2 mm pieces and incubated with 0.2% collagenase (type I, Sigma, St. Louis, MO) with the addition of 350 μl 0.004% DNAase (type I, Sigma)/25 ml collagenase solution. Incubation was carried out at 37°C for 60 min with continuous oxygenation. Cell suspensions were filtered, centrifuged at 175 *g* for 5 min, washed and centrifuged twice in RPMI 1640 solution to remove collagenase. Aliquots (1 ml) of the final tumour cell suspensions were seeded on collagen-coated (Collagen type I, Collaborative Research, Lexington, MA) tissue-culture plates (1.9 $cm^2$). Cell suspensions were carefully mixed before seeding to achieve an even cell density in all wells. However, the seeding densities varied slightly between different experiments, but were always kept between $10^5$ and $10^6$ cells per well. RPMI 1640 culture medium was supplemented with 4% heat-inactivated foetal calf serum, L-glutamine (5 mM), transferrin (5 μg $ml^{-1}$), insulin (5 μg $ml^{-1}$), penicillin (200 IU $ml^{-1}$) and streptomycin (200 μg $ml^{-1}$), and incubated at 37°C in a 90%-humidified atmosphere with 20% $O_2$ and 5% $CO_2$. Tumour cells were grown for 9–13 weeks and media were changed every 3–4 days. Samples of culture media were withdrawn regularly and assayed for 5-HT and NPK-like immunoreactivity (NPK-LI).

*Determination of 5-HT*

Aliquots (10 μl) of culture medium, or peripheral blood after hemolysis and protein precipitation (*cf.* Ahlman *et al.*, 1985), were injected onto the column of an HPLC system with electrochemical detection to determine 5-HT. Standard curves were made by injecting standard solutions of 5-HT (5-hydroxytryptamine-creatininesulfate, Sigma) in 10 μl of 0.1 M perchloric acid (Ponzio & Jonsson, 1978).

*Assay of NPK-LI*

Tachykinins other than substance P were determined by radioimmunoassay using antiserum K12 as previously described (Theodorsson-Norheim *et al.*, 1985). Using the crossreactivity to neurokinin A (NKA) as the 100% reference, the crossreactivity to kassinin was 84%, eledoisin 30%, NKB 26% and NPK 61%. The major immunoreactive component measured by antiserum K12 in media from cultured carcinoid cells is NPK, and NPK-LI will, therefore, subsequently be used to denote the immunoreactive material. In plasma and extracts of tumour tissue antiserum K12 detects NKA, NKA-sulphoxide, NKA (3–10) and NKA (4–10) and an eledoisin-like peptide (Theodorsson-Norheim *et al.*, 1985; Norheim *et al.*, 1987).

*Assay of DNA*

Cultured tumour cells were sonicated in Tris-LiCl and stored at −20°C until assay. The fluorochrome Hoechst 33258 was added to the samples and DNA-concentrations were measured spectrophotometrically (Labarca & Paigen, 1980).

*Statistical methods*

Given values are mean ± s.e.m. and for significance testing we have used unpaired *t*-test, two tailed.

*Experimental protocol*

Cell cultures of the four tumours (cases I–IV) were incubated with SMS 201-995 (Sandoz, Basel, Switzerland) after 4–6 weeks of primary culture. Culture media were changed every 3–4 days and replaced with fresh media containing SMS. During experiments medium concentrations of 5-HT and/or NPK-LI were followed at regular intervals and compared with control cultures in standard medium.

Six experimental protocols were used:

*Protocol 1* Tumour cell cultures (case I) were incubated with two different concentrations of SMS ($10^{-10}$ M or $10^{-8}$ M) during a 2-week period and then allowed to recover for 3 weeks (Figure 2).

*Protocol 2* Tumour cell cultures (case II) were incubated with SMS $10^{-10}$ M for 2 weeks, followed by SMS $10^{-9}$ M for 2 weeks, and finally by SMS $10^{-8}$ M for another 2 weeks. Tumour cells were then allowed to recover for 1–2 weeks (Figure 3).

**Figure 1** Fluorescence micrographs showing human midgut carcinoid tumour cells (case IV) in culture. Tumour cells are strongly labelled by serotonin antibodies **a**, and tachykinin antiserum **b**. The fluorescent material is concentrated to the cytoplasm of tumour cells. Bar indicates 20 μm.

**Figure 2** Medium levels of 5-HT **a**, and NPK-LI **b**, after incubation of tumour cell cultures (case I) with SMS at two different concentrations: $10^{-8}$ M (●) or $10^{-10}$ M (■). Both concentrations caused a significant ($P<0.01$) decrease in 5-HT levels compared with untreated controls (○). The higher concentration also caused a significant reduction in NPK-LI levels ($P<0.05$). Values are given as mean±s.e.m., $n=9$.

**Figure 3** Medium levels of 5-HT **a**, and NPK-LI **b**, after incubation of tumour cell cultures (case II) with SMS 201-995 in increasing concentrations during three successive 2-week periods. SMS treatment cultures (●) had significantly lower levels of both 5-HT and NPK-LI ($P<0.05$) compared with untreated controls (○). Values are given as mean±s.e.m., $n=9-27$.

*Protocol 3* Tumour cell cultures (case III) were incubated with SMS $10^{-8}$ M or the SMS vehicle in the same dilution (acetic acid 2 mg, sodium acetate trihydrate 2 mg, sodium chloride 7 mg and 1 ml of sterile water) for 5 weeks. Addition of the diluted vehicle did not affect the pH of the culture media (Figure 4).

*Protocol 4* Tumour cell cultures (case III) were incubated with SMS $10^{-10}$ M or $10^{-8}$ M during 2 weeks and the DNA contents in the cell cultures were determined at the end of the experiments (Table I).

*Protocol 5* Tumour cell cultures (case III) were incubated with SMS $10^{-8}$ M for 4 days. At the end of the experiments the cultures were sonicated and the intracellular contents of 5-HT and DNA were determined (Figure 5).

*Protocol 6* The β-adrenoceptor induced release of 5-HT was studied in tumour cell cultures (cases III and IV) after short-term incubation (5 min) with isoprenaline (IP) $10^{-6}$ M. These cultures were pretreated with SMS $10^{-8}$ M for 1–4 h, or for 4 days (Figure 6).

### *Microscopy*

Tumour cell cultures were examined and photographed at regular intervals using a phase contrast microscope (Nikon-Diaphot). The presence of 5-HT and tachykinins in tumour cells were studied by immunofluorescence. Cell cultures were fixed in 4% paraformaldehyde in PBS (pH 7.4) and incubated with anti-5-HT antibodies (1:200 YC 5/45 HKL; Sera-Lab Ltd, Crawley Down, Sussex, UK) or tachykinin antiserum NKA2 1: 500 (Brodin *et al.*, 1986). Antibody binding sites were visualised using a biotin-streptavidin system (Vector Lab, Burlingame, CA). Controls included substitution of the specific antiserum for normal serum of corresponding species.

**Figure 4** Medium levels of 5-HT after incubation of tumour cell cultures (case III) with SMS 201-995 ($10^{-8}$ M) (●) or the SMS vehicle) (○). SMS caused a significant decrease in 5-HT concentrations ($P<0.05$). Both groups showed an interesting cyclic variation in 5-HT levels. Values are given as mean±s.e.m., $n=12$.

**Table I** Effect of SMS treatment during 2 weeks on 5-HT in medium and DNA contents of human midgut carcinoid tumour cells in culture

| | *DNA contents (μg well)* | *5-HT in medium (nmol l⁻¹)* | *Ratio 5-HT:DNA* |
|---|---|---|---|
| **(A)** | | | |
| Controls | 5.44±0.66 | 757±64.5 | 170.5±10.2 |
| SMS $10^{-10}$ M | 5.10±0.74 | 641±18.4 | 154.8±20.8 |
| **(B)** | | | |
| Controls | 17.4±1.5 | 2221±95.1 | 133.6±7.2 |
| SMS $10^{-8}$ M | 17.5±0.90 | 1477±118.9[a] | 85.8±7.8[a] |

Values are given as mean±s.e.m., $n=12$; [a]$P<0.001$.

**Figure 5** Tumour cell cultures (case III) were incubated with SMS 201-995 ($10^{-8}$ M) during 4 days. The levels of 5-HT in the medium **a**, and intracellular 5-HT **b**, as well as the DNA contents in the cultures **c**, were studied. When SMS treated cultures (■) were compared with untreated cultures (▨), even this short period caused a significant ($P<0.001$) reduction of both the medium levels and the intracellular levels of 5-HT. The DNA contents increased over 4 days, but was not affected by SMS treatment. Values are given as mean ± s.e.m., $n = 6-24$.

**Figure 6** **a**, Tumour cell cultures (case III) were stimulated with isoprenaline (IP $10^{-6}$ M) 1 (○) or 4 days (□) after change of media. The cells stimulated 4 days after media change had significantly higher media levels of 5-HT, but the release pattern of 5-HT upon IP stimulation was very similar. Pretreatment with SMS ($10^{-8}$ M) (■) for 4 days kept the basal levels of 5-HT low but did not affect the release after IP stimulation. Values are given as mean ± s.e.m., $n = 8-24$. **b**, Tumour cell cultures (case IV) were stimulated with IP ($10^{-6}$ M) 1 h (●) or 4 h (■) after change of media and addition of SMS ($10^{-8}$ M) and were compared with unstimulated controls (○ 1 h, □ 4 h). The IP stimulated release was not prevented by SMS. Values are given as mean ± s.e.m., $n = 4-8$.

### Results

*Influence of SMS 201-995 on the levels of 5-HT and NPK-LI and on the DNA contents of tumour cell cultures.*

Cultured tumour cells from all four patients were positively labelled with 5-HT antibodies and the tachykinin antiserum. Intense labelling was observed over the cytoplasm of tumour cells (Figure 1).

Incubation of tumour cells with two different concentrations of SMS ($10^{-10}$ M or $10^{-8}$ M) for a 2-week period (protocol 1) caused a marked decrease in the 5-HT levels in culture media. Treatment with SMS $10^{-8}$ M, but not $10^{-10}$ M, also caused a significant ($P<0.05$) reduction in the levels of NPK-LI. Two weeks after cessation of SMS treatment the levels of 5-HT and NPK-LI in culture media were similar to those in untreated controls (Figure 2a,b).

Tumour cells, incubated with increasing concentrations of SMS ($10^{-10}$ M–$10^{-8}$ M) during three successive 2-week periods (protocol 2), maintained the concentrations of 5-HT and NPK-LI at a low level compared with non-treated controls. Even 2 weeks after the test period tumour cell culture media had significantly lower levels of 5-HT than controls, while the levels of NPK-LI did not differ. At the end of the observation period the 5-HT levels of controls were still high while the NPK-LI levels were much reduced (Figure 3a,b).

Tumour cells incubated with SMS $10^{-8}$ M over 5 weeks (protocol 3) showed much lower 5-HT levels than tumour cells incubated with the SMS vehicle alone during the entire stimulation period. Both groups showed a cyclic variation (with 15 day cycles) of 5-HT levels in the media, as previously observed in long-term cultures (Åhlund *et al.*, 1989*a*). No signs of densensitisation to SMS were noted (Figure 4).

Incubation of tumour cell cultures with SMS ($10^{-10}$ M or $10^{-8}$ M) during 2 weeks (protocol 4) resulted in significantly decreased 5-HT levels compared with untreated controls. However, the DNA contents were very similar in treated and non-treated groups (Table I). Cells from the same tumour were seeded at two different cell densities. A constant, but low, number of fibroblasts were demonstrated in all cultures. The ratio of 5-HT concentration in culture media over DNA contents of the two types of cultures did not differ. However, this ratio was clearly suppressed by SMS treatment (Table I). Tumour cell cultures from case III were also treated with SMS ($10^{-8}$ M) during 4 days (protocol 5). Even this short treatment significantly reduced the media levels of 5-HT, as well as the intracellular levels of 5-HT, detected after sonication of the cultures. The DNA contents in these experiments were also similar in treated and non-treated cultures (Figure 5a,b,c).

*Influence of SMS 201-995 on β-adrenoceptor induced release of 5-HT*

Tumour cell cultures from case III were stimulated with IP ($10^{-6}$ M) 1 or 4 days after change of media. In both situations a pronounced release of 5-HT was demonstrated. However, the cells studied after 4 days had significantly higher media levels of 5-HT. Pretreatment with SMS $10^{-8}$ M for 4 days kept the basal 5-HT concentration in the media at a similar level as at onset of the experiment. The release of 5-HT upon IP stimulation ($10^{-6}$ M) was, however, similar to untreated controls (Figure 6a).

Tumour cell cultures from case IV (protocol 5) also showed a pronounced release of 5-HT with IP ($10^{-6}$ M) after pretreatment with SMS $10^{-8}$ M for 1 or 4 h. Sole incubation with SMS $10^{-8}$ M during these time periods had no effect on the 5-HT levels (Figure 6b).

### Discussion

Somatostatin was originally characterised as a peptide hormone (14 amino acids) inhibiting the release of growth hormone in the hypothalamus (Brazeau *et al.*, 1973). However, somatostatin occurs widely in the CNS and in the gastro-enteric-pancreatic endocrine system. It has been ascribed a role as physiological regulator of secretion, e.g. it inhibits the secretion of pancreatic and gut hormones and exocrine secretion as well (Reichlin, 1983). Bauer *et al.* (1982) synthesised an analogue (SMS 201-995) of the conformationally stable part of somatostatin (eight amino acids), which was highly resistant to degradation and selective in its inhibition of growth hormone secretion. This compound has become a most valuable medical adjunct in the treatment of several pancreatic and gut endocrine tumours due to its suppression of hormone overproduction (Lamberts *et al.*, 1987*a*; Lamberts *et al.*, 1990).

In the present study we have obtained evidence for a direct effect of SMS on human midgut carcinoid tumours in culture. In all four tumours studied SMS reduced the media levels of both 5-HT and NPK-LI in cultures of human midgut carcinoid tumour cells within 4 days. The reduction appeared to be stable during the period of study and was similar at the two concentrations tested. In one experimental protocol (2) control tumour cells were observed over 90 days. The low media levels of NPK-LI at the end of this period contrasted with well maintained levels of 5-HT. This finding might indicate that the synthesis and secretion of peptides and amines have different control mechanisms. Such differences are evident in the response to stimulation with β-adrenoceptor agonists, i.e. 5-HT being released without changes of tachykinin levels (Ahlman *et al.*, 1988*b*). In these experiments pretreatment of tumour cells with SMS did not significantly inhibit the β-adrenoceptor induced release of 5-HT, tested after pretreatment up to 4 days. This is in contrast to the clinical situation, where treatment with SMS for 4 days reducts both the basal and provoked levels of 5-HT (Ahlman *et al.*, 1988*a*). Case I in this series had decreased levels of both urinary 5-HIAA (63%) and basal 5-HT in peripheral blood (63%) accompanied by an extinguished release reaction to PG after 4 weeks of treatment with SMS. Case II had decreased 5-HIAA levels (23%) and no release reaction upon PG provocation after 3 months of treatment. Both these patients underwent similar treatment with surgical debulking, liver ischemia and continuous SMS treatment leading to a long-lasting state of biochemical normalisation. Sole treatment with SMS in the carcinoid syndrome had not been reported to reduce 5-HIAA levels into the normal range of any patients (Gorden *et al.*, 1989). Case II and IV underwent uneventful major surgical procedures under protection with SMS according to a previously reported program (Ahlman *et al.*, 1988*a*) resulting in total remission of disease radiologically as well as biochemically.

PG has previously been shown not to cause release of 5-HT from human midgut carcinoid tumour cells *in vitro* (Nilsson *et al.*, 1985). *In vivo* there is strong experimental evidence that PG acts via an indirect mechanism causing release of catecholamines from the adrenals, in turn activating β-adrenoceptors located on enterochromaffin cells (or carcinoid tumour cells) (Grönstad *et al.*, 1987). Previous studies using autoradiography and *in vitro* binding assay have shown saturable and high affinity receptors with specificity for somatostatin on several different human endocrine tumours (Reubi *et al.*, 1987*a,b*; Lamberts *et al.*, 1990). Our findings indicate that SMS, presumably bound to receptors on the midgut carcinoid tumour cells, inhibits the secretion of tumour products via a second messenger system different from the one mediating the β-adrenoceptor stimulated release of 5-HT. The discrepancy between the blocked PG response seen clinically and the unaffected release of 5-HT at β-adrenoceptor stimulation seen *in vitro* (e.g. Case III) may indicate that SMS also reduces the PG induced release of catecholamines from the adrenal medulla. Alternatively, the β-adrenoceptor mechanism may operate via a modified tumour receptor, since the IP-induced release from tumour cell cultures cannot be blocked by β-adrenoceptor antagonists (Ahlman *et al.*, 1988*b*). However, immunocytochemically, midgut carcinoid tumour cells display a positive reaction with an antiserum directed against the β-adrenoceptor protein (Wängberg *et al.*, 1990). The decreased intracellular levels of 5-HT after short-term (4 days) treatment with SMS, in combination with unchanged DNA contents, may indicate a suppressed synthesis of 5-HT as well. In the present study we have confirmed our previous observations (Åhlund *et al.*, 1989*a*) on a cyclicity of 5-HT secretion from cultured carcinoid tumour cells (Figures 2 and 4). Parallel changes in the secretion of NPK-LI were also observed (Figure 2) and SMS treatment did not abolish the cyclicity. Since the assays were done in separate laboratories and at different times a technical error is less likely. Periodic induction of enzyme systems by local production of certain factors cannot be excluded.

Naturally occurring somatostatin appears to be an endogenous growth inhibitor, which delays the separation of centrosomes indicative of the G1 phase (Mascardo & Sherline, 1982). With the recent development of potent somatostatin analogues a certain interest has been focused on the inhibition of growth of experimental tumours in animal models i.e. rat chondrosarcomas (Reubi, 1985), mice osteosarcoma (Cai *et al.*, 1986), acinar pancreatic carcinoma in rats (Redding & Schally, 1984), prolactin-secreting pituitary carcinoma in rats (Lamberts *et al.*, 1986), and mammary, prostate and ductal pancreatic carcinomas in rats (Cai *et al.*, 1986). Several mechanisms have been proposed e.g. inhibition of the local production of growth factors, inhibition of growth hormone and somatomedin C, specific binding to tumour cell receptors with subsequent interference of intracellular signals, or inhibition of the effects of oncogene products (*cf.* Lamberts *et al.*, 1987*a*; Schally, 1988). The antiproliferative effect of somatostatin was studied by Mascardo and Sherline (1982) on two different cell lines and was found to be effective in the range $10^{-13}-10^{-8}$ M, coupled with the physiological inhibition of secretory processes. Previous experiments on cell cultures from an oestrogen-induced, transplantable rat pituitary carcinoma have demonstrated an inhibitory effect of SMS ($10^{-9}$ M) on both basal and somatomedin C-stimulated secretion of prolactin and on cell proliferation (Lamberts *et al.*, 1986). Somatostatin was further compared with two analogues in the inhibition of proliferation of a human breast cancer cell line and maximal effects were observed at the $10^{-9}$ M concentration.

In the present experiments the DNA contents of human midgut carcinoid tumour cells were studied after 4 days or 2 weeks of incubation with SMS ($10^{-10}$ and $10^{-8}$ M). Within these periods of time the SMS treatment had caused a pronounced reduction of the media levels of 5-HT without any observable effect on the DNA contents of the tumour cell cultures. In the phase contrast microscope tumour cells treated with SMS could not be distinguished from untreated cells, and the density of tumour cells appeared unchanged. The reduction in 5-HT levels observed during SMS treatment is therefore most likely due to an inhibition of hormone secretion, and possibly reduced hormone synthesis, from the tumour cells rather than unchanged release from a reduced number of tumour cells. In clinical studies on patients with the midgut carcinoid syndrome, using SMS treatment, antiproliferative effects have been reported only in few patients in large series (Soquet *et al.*, 1987; Ahlman *et al.*, 1991; Gorden *et al.*, 1989) in line with the present experimental findings.

We wish to thank Mss A. Wigander, L. Johansson, K. Lundmark and A.-K. Illerskog for expert technical assistance. This study was supported by the Swedish MRC (2207,5220), Swedish Cancer Society (2998-B91-01X), I-B & A. Lundberg's Research Foundation, T & R Söderbergs Foundation, the Swedish Medical Society, A. Gabrielsson's Foundation, Jubileumsklinikens Cancer Research Fund, the Nordic Foundation for Scientific Research without Animal Experiments and the Göteborg Medical Society.

### References

AHLMAN, H., DAHLSTRÖM, A., GRÖNSTAD, K. & 4 others (1985). The pentagastrin test in the diagnosis of the carcinoid syndrome. Blockade of gastrointestinal symptoms by ketanserin. *Ann. Surg.*, **201**, 81.

AHLMAN, H., ÅHLUND, L., DAHLSTRÖM, A., MARTNER, J., STENQVIST, O. & TYLÉN, U. (1988*a*). SMS 201-995 and provocation tests in preparation of patients with carcinoids for surgery or hepatic arterial embolisation. *Anesth. Analg.*, **67**, 1142.

AHLMAN, H., ÅHLUND, L., NILSSON, O., SKOLNIK, G., THEODORSSON, E. & DAHLSTRÖM, A. (1988*b*). Carcinoid tumour cells in long-term culture: release of serotonin but not of tachykinins on stimulation with adrenoceptor agonists. *Int. J. Cancer*, **42**, 506.

AHLMAN, H., WÄNGBERG, B., JANSSON, S. & 6 others (1991). Management of disseminated midgut carcinoid tumours. *Digestion* (in press).

ÅHLUND, L., NILSSON, O., KLING-PEDERSEN, T. & 4 others (1989*a*). Serotonin-producing carcinoid tumour cells in long-term culture. Studies on serotonin release and morphological features. *Acta Oncol.*, **28**, 341.

ÅHLUND, L., KINDBLOM, L.G., NILSSON, O. & 4 others (1989*b*). Clinical and experimental studies on a midgut carcinoid tumor. *J. Surg. Onc.*, **41**, 86.

BAUER, W., BRINER, U., DOEPFNER, W. & 5 others (1982). SMS-201-995: a very potent and selective octapeptide analogue of somatostatin with prolonged action. *Life. Sci.*, **31**, 1133.

BRAZEAU, P., VALE, W., BURGUS, R. & 4 others (1973). Hypothalamic polypeptide that inhibits the secretion of immunoreactive pituitary growth hormone. *Science*, **179**, 77.

BRODIN, E., LINDEFORS, N., THEODORSSON-NORHEIM, E. & ROSELL, S. (1986). Tachykinin multiplicity in rat central nervous system as studied using antisera raised against substance P and neurokinin A. *Regulatory Peptides*, **13**, 253.

CAI, R.Z., SZOKE, B., LU, R., FU, D., REDDING, T.W. & SCHALLY, A.V. (1986). Synthesis and biological activity of highly potent octapeptide analogs of somatostatin. *Proc. Natl Acad. Sci. USA*, **83**, 1896.

GORDEN, P., COMI, R.J., MATON, P.N. & GO, V.L.W. (1989). Somatostatin and somatostatin analogue (SMS 201-995) in treatment of hormone-secreting tumors of the pituitary and gastrointestinal tract and non-neoplastic diseases of the gut. *Ann. Intern. Med.*, **110**, 35.

GRÖNSTAD, K.O., NILSSON, O., DAHLSTRÖM, A., SKOLNIK, G. & AHLMAN, H. (1987). Adrenergic control of serotonin release from human carcinoid tumour cells *in vitro* and *in vivo*. *J. Surg. Res.*, **42**, 141.

KVOLS, L.K., MARTIN, J.K., MASH, H.M. & MOERTEL, C.G. (1985). Rapid reversal of carcinoid crisis with a somatostatin analogue. *N. Engl. J. Med.*, **313**, 1229.

KVOLS, L.K., MOERTEL, C.G., O'CONNELL, M.J., SCHUTT, A.J., RUBIN, J. & HAHN, R.G. (1986). Treatment of the malignant carcinoid syndrome: evaluation of a long acting somatostatin analogue. *N. Engl. J. Med.*, **315**, 663.

LABARCA, C. & PAIGEN, K. (1980). A simple, rapid and sensitive DNA assay procedure. *Analyt. Biochem.*, **102**, 344.

LAMBERTS, S.W.J., REUBI, J.C., UITTERLINDEN, P., ZUIDERWIJK, J. VAN WERFF, P. & VAN HAL, P. (1986). Studies on the mechanism of action of the inhibitory effect of the somatostatin analogue SMS 201-995 on the growth of the PRL/ACTH pituitary tumor 7315a. *Endocrinology*, **118**, 2188.

LAMBERTS, S.W.J., KOPER, J.W. & REUBI, J.C. (1987*a*). Potential role of somatostatin analogues in the treatment of cancer. *Eur. J. Clin. Invest.*, **17**, 281.

LAMBERTS, S.W.J., VERLEUN, T., ZUIDERWIJK, J.M. & OOSTEROM, R. (1987*b*). The effect of the somatostatin analogue SMS 201-995 on normal growth hormone secretion in the rat. A comparison with the effect of bromocriptine on normal prolactin secretion. *Acta Endocrinol.*, **115**, 196.

LAMBERTS, S.W.J., HOFLAND, L.J., VAN KOETSVELD, P.M. & 4 others (1990). Parallel *in vivo* and *in vitro* detection of functional somatostatin receptors in human endocrine pancreatic tumors: consequences with regard to diagnosis, localization and therapy. *J. Clin. Endocrinol. Metab.*, **71**, 566.

NILSSON, O., GRÖNSTAD, K.O., GOLDSTEIN, M., SKOLNIK, G., DAHLSTRÖM, A. & AHLMAN, H. (1985). Adrenergic control of 5-HT release from a midgut carcinoid tumor. *Int. J. Cancer*, **36**, 307.

MASCARDO, R.N. & SHERLINE, P. (1982). Somatostatin inhibits rapid centrosomal separation and cell proliferation induced by epidermal growth factor. *Endocrinology*, **111**, 1394.

NORHEIM, I., WILANDER, E., ÖBERG, K. & 4 others (1987). Tachykinin production by carcinoid tumours in culture. *Europ. J. Cancer*, **23**, 689.

ÖBERG, K., NORHEIM, I., THEODORSSON, E., AHLMAN, H., LUNDQVIST, G. & WIDE, L. (1989). The effect of octreotide on basal and stimulated hormone levels in patients with carcinoid syndrome. *J. Clin. Endocrinol. Metab.*, **68**, 796.

PONZIO, F. & JONSSON, G. (1978). A rapid and simple method for the determination of picogram levels of serotonin in brain tissue using liquid chromatography with electrochemical detection. *J. Neurochem.*, **32**, 129.

REDDING, T.W. & SCHALLY, A.V. (1984). Inhibition of growth of pancreatic carcinomas in animal models by analogs of hypothalamic hormones. *Proc. Natl Acad. Sci. USA*, **81**, 248.

REICHLIN, S. (1983). Somatostatin. *N. Engl. J. Med.*, **309**, 1495, 1556.

REUBI, J.C.A. (1985). A somatostatin analogue inhibits chondrosarcoma and insulinoma tumour growth. *Acta Endocrinol.*, **109**, 108.

REUBI, J.C., MAURER, R., VON WERDER, K., TORHORST, J., KLIJN, J.G.M. & LAMBERTS, S.W.J. (1987*a*). Somatostatin receptors in human endocrine tumors. *Cancer Res.*, **47**, 551.

REUBI, J.C., HÄCKI, W.H. & LAMBERTS, S.W.J. (1987*b*). Hormone-producing gastrointestinal tumors contain a high density of somatostatin receptors. *J. Clin. Endocrinol. Metab.*, **65**, 1127.

SCHALLY, A.V. (1988). Oncological applications of somatostatin analogues. *Cancer Res.*, **48**, 6977.

SOQUET, J.C., SASSOLAS, G., FORICHON, J., CHAMPETIER, P., PARTENSKY, C. & CHAYVIALLE, J.A. (1987). Clinical and hormonal effects of a long acting somatostatin analogue in pancreatic endocrine tumours and in carcinoid syndrome. *Cancer*, **59**, 1654.

THEODORSSON-NORHEIM, E., NORHEIM, I., ÖBERG, K. & 4 others (1985). Neuropeptide K: a major tachykinin in plasma and tumor tissues from carcinoid patients. *Biochem. Biophys. Res. Comm.*, **131**, 77.

VINIK, A. & MOATTARI, A.R. (1989). Use of somatostatin analogue in management of carcinoid syndrome. *Dig. Dis. Sci.*, **34**, 14.

WÄNGBERG, B., AHLMAN, H., NILSSON, O., HAGLID, K., DENNEY, R.M. & DAHLSTRÖM, A. (1990). Amine handling properties of human carcinoid tumour cells in tissue culture. *Neurochem. Int.*, **17**, 331.